# Lipoylation is dependent on the ferredoxin FDX1 and dispensable under hypoxia in human cells

**DOI:** 10.1016/j.jbc.2023.105075

**Published:** 2023-07-20

**Authors:** Pallavi R. Joshi, Shayan Sadre, Xiaoyan A. Guo, Jason G. McCoy, Vamsi K. Mootha

**Affiliations:** 1 Broad Institute, Cambridge, Massachusetts, USA; 2 Department of Molecular Biology, Howard Hughes Medical Institute, Massachusetts General Hospital, Boston, Massachusetts, USA; 3 Department of Systems Biology, Harvard Medical School, Boston, Massachusetts, USA

**Keywords:** mitochondria, iron–sulfur protein, SAM, energy metabolism, hypoxia, lipoate

## Abstract

Iron–sulfur clusters (ISC) are essential cofactors that participate in electron transfer, environmental sensing, and catalysis. Amongst the most ancient ISC-containing proteins are the ferredoxin (FDX) family of electron carriers. Humans have two FDXs- FDX1 and FDX2, both of which are localized to mitochondria, and the latter of which is itself important for ISC synthesis. We have previously shown that hypoxia can eliminate the requirement for some components of the ISC biosynthetic pathway, but FDXs were not included in that study. Here, we report that FDX1, but not FDX2, is dispensable under 1% O_2_ in cultured human cells. We find that FDX1 is essential for production of the lipoic acid cofactor, which is synthesized by the ISC-containing enzyme lipoyl synthase. While hypoxia can rescue the growth phenotype of either FDX1 or lipoyl synthase KO cells, lipoylation in these same cells is not rescued, arguing against an alternative biosynthetic route or salvage pathway for lipoate in hypoxia. Our work reveals the divergent roles of FDX1 and FDX2 in mitochondria, identifies a role for FDX1 in lipoate synthesis, and suggests that loss of lipoic acid can be tolerated under low oxygen tensions in cell culture.

Iron–sulfur clusters (ISCs) are ancient cofactors believed to have first formed in primordial oceans under anaerobic conditions (1, 2). Common forms of ISCs are the 2Fe–2S and 4Fe–4S clusters, which can perform one-electron transfer reactions, catalyze dehydration reactions, activate aliphatic substrates, and stabilize proteins (1, 2, 3). In most eukaryotes, ISCs are assembled *via* the mitochondrial ISC pathway, which begins with the synthesis of a 2Fe–2S followed by incorporation into ISC proteins or reductive coupling with another cluster to form a 4Fe–4S cluster (4, 5, 6). ISC synthesis in mitochondria is initiated by loading a ferrous (Fe^2+^) iron onto the scaffold protein ISCU (4, 7). ISCU associates with the cysteine desulfurase NFS1 and its cofactors that together catalyze the conversion of a cysteine to an alanine and transfer a sulfur group in the form of a persulfide to ISCU in the presence of frataxin (FXN) (4). For sulfur release from the persulfide to make a 1Fe–1S cluster, the input of two electrons is required (4). One of these electrons is donated by the iron itself and the other is donated by the electron carrier ferredoxin 2 (FDX2) (4, 8). To achieve a final 2Fe–2S product, ISCU is believed to dimerize (4, 5).

To date, over 60 ISC-containing human proteins have been discovered that localize to the nucleus, cytosol, or mitochondria (7, 9). In the cytosol and nucleus, ISC proteins participate in reactions such as the breakdown of xanthine (XDH) and synthesis of the molybdenum cofactor (MOCS1A), or DNA maintenance (MUTYH, NTHL1) and replication (POLD1, POLE1) (10, 11, 12). In mitochondria, ISC-containing proteins include subunits of complexes I (CI), II (CII), and III (CIII) of oxidative phosphorylation (OXPHOS), lipoyl synthase (LIAS), as well as FDXs (3, 5, 13).

FDXs are versatile single electron carriers found in all domains of life (14, 15). Because the midpoint potential of their ISC is finely tuned by the local protein environment and solvent exposure, organisms can harbor multiple FDXs that simultaneously function in distinct cellular reactions (14, 16). Humans and other chordates have two FDXs (FDX1 and FDX2), both of which contain a 2Fe–2S cluster (17), localize to the mitochondrial matrix (17), and are reported to receive electrons from a mitochondrial NADPH-dependent ferredoxin reductase (FDXR) (17, 18, 19). Foundational studies ascribed specific roles for the two FDXs (Fig. 1*A*), with FDX1 functioning primarily in sterol synthesis pathways by donating electrons to various cytochrome P450 proteins such as CYP11A1 (19, 20), and FDX2 functioning in the more ancient role of electron donation to the ISC machinery as well as the synthesis of heme A, which is required for complex IV (CIV) of the electron transport chain (8, 17, 21, 22) (Fig. 1*A*). Whether FDXs contribute to other mitochondrial pathways remains an open question (Fig. 1*A*).

**Figure 1:**
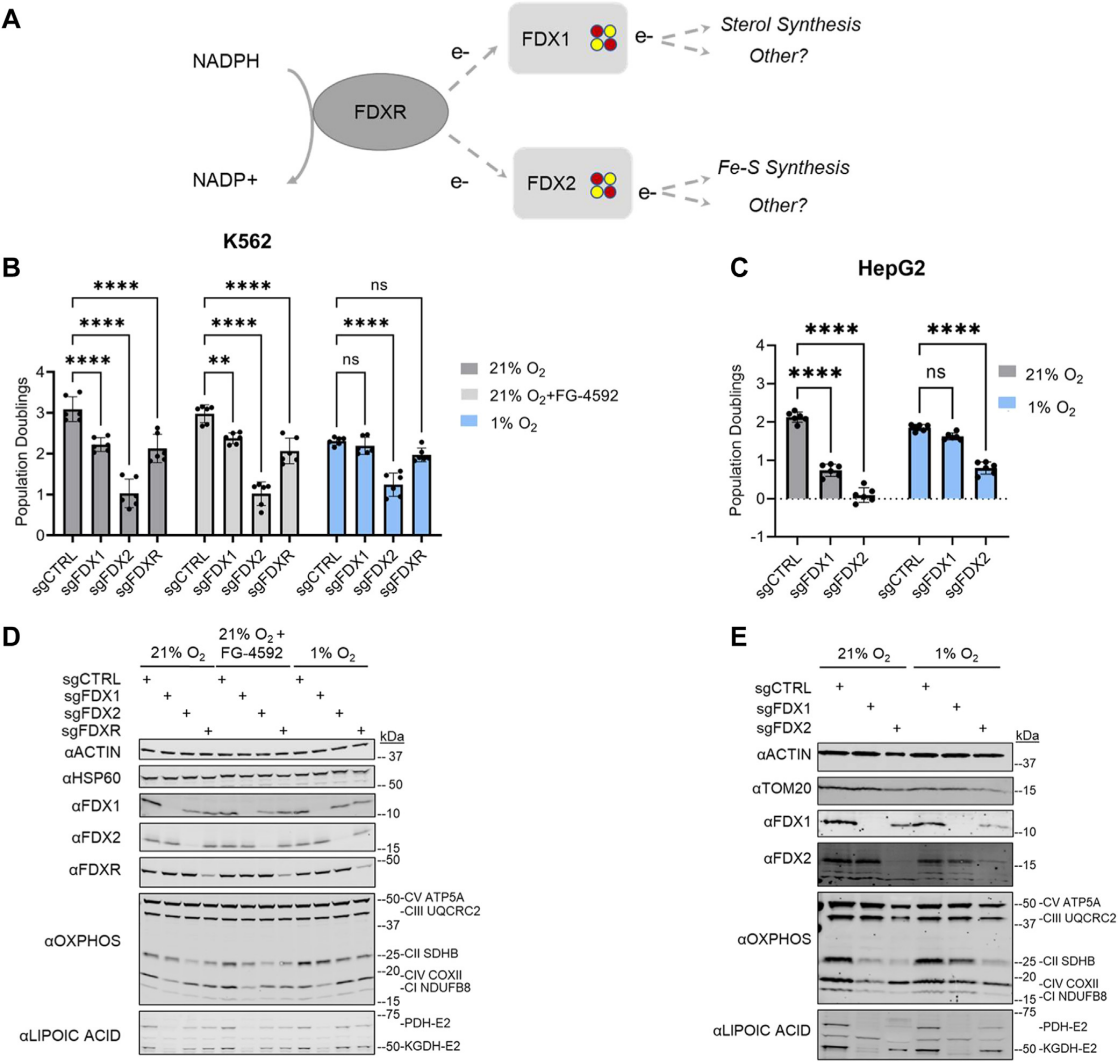
**FDX1, but not FDX2, is dispensable for growth in hypoxic conditions.***A*, current model of human FDXs and their main functions. *B*, three-day proliferation assay of K562 cells edited with control (CTRL), *FDXR*, *FDX1*, or *FDX2* CRISPR guides. Cells were grown in 21% O_2_, 1% O_2_, or treated with 75 μM of HIF-activator FG-4592 in 21% O_2_. *C*, three-day proliferation assay of HepG2 cells edited with control, *FDX2*, or *FDX1* guides and grown in 21% or 1% O_2_. *D*, immunoblots for FDXR, FDX1, FDX2, select OXPHOS subunits, lipoylated PDH and KGDH, and control proteins ACTIN and HSP60 on lysates of edited K562 cells used for proliferation assay. *E*, immunoblots for FDX1, FDX2, select OXPHOS subunits, lipoylated PDH and KGDH, and control proteins ACTIN and TOM20 on lysates of edited HepG2 cells used for proliferation assay. All bar plots show mean ± SD of three independent experiments. ns = *p* > 0.05, ∗*p* ≤ 0.05, ∗∗*p* ≤ 0.01, ∗∗∗*p* ≤ 0.001, ∗∗∗∗*p* ≤ 0.0001. Two-way ANOVA with Bonferroni’s post-test. FDX, ferredoxin; HIF, hypoxia-inducible factor; KGDH, α-ketoglutarate dehydrogenase; OXPHOS, oxidative phosphorylation; PDH, pyruvate dehydrogenase.

Previous work from our laboratory has demonstrated in yeast, worms, and human cells that loss of the ISC machinery protein FXN, which is mutated in Friedreich’s ataxia, can be buffered by hypoxia (23). In this same study, we found that much of the brain pathology of an shFXN mouse model of Friedreich’s ataxia could also be prevented when mice are breathing 11% oxygen (23). In addition, we discovered that core ISC biosynthetic machinery (such as NFS1 and ISCU) is always essential, but the electron donor systems, FDX2 and FDXR, were not tested (23). A subsequent low/high oxygen CRISPR screen from our laboratory broadened the spectrum of mitochondrial proteins, which, like FXN, may be dispensable under low oxygen tensions (24). The screening results confirmed the dispensability of FXN, but no other core ISC components scored, including FDX2 or FDXR (24). Curiously, the screen did uncover FDX1 as potentially dispensable in low O_2_ (24). An earlier genome-wide CRISPR galactose death screen designed to discover proteins required for oxidative phosphorylation identified both FDX1 and FDXR but not FDX2 (25). A recent report implicated FDX1 as an upstream regulator of LIAS (26), which generates the lipoic acid cofactor required for many tricarboxylic acid (TCA) cycle enzymes including pyruvate dehydrogenase (PDH) (27, 28, 29).

The combined evidence from these studies motivated us to investigate the roles of FDX1, FDX2, and FDXR in ISC synthesis and lipoate metabolism, as well as to evaluate their requirements under hypoxia. We also find that FDX1 is required for lipoate synthesis, and in addition, report that FDX1, but not FDX2, is dispensable under low oxygen tensions. Surprisingly, lipoate levels are not rescued in either *FDX1* or *LIAS* KO cells under hypoxia. Hence, the loss of lipoate appears to be tolerated in low oxygen tensions in cultured human cells.

## Results

### FDX1, but not FDX2, is dispensable for growth in hypoxia

We began by testing whether FDXR, FDX1, or FDX2 are dispensable in low oxygen. While our prior study showed that FXN was unique amongst tested ISC assembly machinery components, we did not assess FDXR and FDX2 at that time (23). Although these two proteins did not score in our previous low/high O_2_ screen, FDX1 did (ranking 96 amongst 20,113 targeted genes) (24). We thus utilized CRISPR/Cas9-mediated gene editing to knockout *FDX1*, *FDX2*, or *FDXR* in K562 cells. At 21% O_2_ (normoxia), the cells grew with varying growth defects (Fig. 1*B*). However, when cells were grown continuously in 1% O_2_ (hypoxia), FDX1 and FDXR KO growth rate was similar to the control KO cells, although FDXR KO cells still showed a mild deficit (Fig. 1*B*).

A natural question is whether the rescue by hypoxia is mediated by the hypoxia-inducible factor (HIF) transcriptional response, which is activated by low oxygen and upregulates numerous pathways required for cell survival under hypoxia (30). In normoxia, HIF is hydroxylated by the prolyl hydroxylase enzymes (prolyl hydroxylase domain) and subsequently ubiquitinated by VHL, which targets the protein for proteasomal degradation (30, 31). We previously showed that HIF activation, unlike hypoxia, was not sufficient to rescue *FXN KO* cell growth (23). We therefore treated our control and KO cells with the prolyl hydroxylase domain inhibitor FG-4592 in normoxic conditions (Fig. 1*B*) (32). Expression of the canonical HIF target *BNIP3L* was indeed increased by this drug as confirmed by quantitative PCR (qPCR) (Fig. S1) (33), but the drug was not sufficient to ameliorate the growth defects of our *FDXR*, *FDX1*, and *FDX2* KO K562 cell lines (Fig. 1*B*).

We could extend this observation to a second cell type, HepG2, which unlike K562 cells in our hands did not exhibit a baseline growth defect in 1% O_2_. We repeated our growth assay experiments with control, *FDX1*, and *FDX2* KO cells in 21% and 1% oxygen tensions and once again found that FDX1, but not FDX2, was dispensable under low oxygen tensions, whereas FDXR KO growth was also rescued in this cell line (Figs. 1*C* and S2). HepG2 cells lacking FDX2 were not viable in normoxia, as could be seen by the global depletion of proteins and large aggregates visible by Ponceau staining of nitrocellulose membranes (Fig. S3); in hypoxia, these phenotypes were milder and FDX2 loss was more tolerable, although the cells still grew significantly slower than controls (Figs. 1*C* and S3).

Collectively, these studies demonstrate that FDX1 is dispensable in two different cell types when grown in hypoxic conditions, and that this effect cannot be recapitulated by forced stabilization of HIF in K562 cells. In contrast, *FDX2* KO cell phenotypes were in-line with previous findings that knockouts of core ISC biosynthetic genes *NFS1*, *ISCU*, and *LYRM4* are not rescued by hypoxia (23).

### Contrary to growth, lipoate depletion in FDX1 KO cells is not rescued by hypoxia

Given that hypoxia rescues FXN deficiency in an HIF-independent manner by restoring ISC levels (23), we asked if hypoxia can also restore the biochemical defects in *FDX1* KOs by a similar mechanism. We performed immunoblotting on cell lysates and sought to determine whether the knockouts exhibited biochemical phenotypes of ISC deficiency within the mitochondria, such as the loss of ISC-containing respiratory chain complexes CI and CII, or a reduction in lipoate synthesis because of loss of the ISC-containing LIAS protein, and whether these defects could be restored under low O_2_ (5, 7, 23, 34, 35).

Knockout of *FDX1* or *FDX2* in either cell line resulted in loss of CI, CII, and CIV to varying degrees (Fig. 1, *D* and *E*). Surprisingly, while FDX2 purportedly has a role in the synthesis of heme A, the cofactor found in CIV (17, 21), FDX1 loss caused a greater depletion of CIV, which was rescued in HepG2 but not K562 cells under hypoxia (Fig. 1, *D* and *E*). A recent study published during the preparation of this report confirmed that FDX1 plays a role in heme A synthesis, although the exact degree of FDX2 involvement remains unclear (36). Additional deficiencies in CI and CII were also seen with FDX1 and FDX2 loss and rescued under hypoxia in *FDX1* (and not *FDX2*) KO HepG2 cells (Fig. 1, *D* and *E*). The addition of FG-4592 to KO K562 cells did not rescue any of these defects seen in 21% O_2_ (Fig. 1*D*).

We next performed immunoblotting for lipoate. Four enzymes in the TCA cycle (PDH, α-ketoglutarate dehydrogenase [KGDH], branched-chain α-ketoacid dehydrogenase, and 2-oxoadipate dehydrogenase) as well as the glycine cleavage system H protein use lipoic acid as a cofactor (27), and simultaneous immunoblotting for lipoylation of PDH and KGDH can be used as a readout of lipoate steady-state levels (37, 38). *FXN* KO cells have reduced lipoylation of PDH and KGDH, and this deficit was restored under hypoxia because of the restoration of ISC availability (23). Recent reports have indicated that loss of FDX1 leads to near complete ablation of PDH and KGDH lipoylation (26, 36). We confirmed these results in both K562 and HepG2 cells (Fig. 1, *D* and *E*). We in addition found in our knockouts that lipoate levels were more depleted in *FDX1* KO cells compared with the *FDXR* or *FDX2* KO samples (Fig. 1, *D* and *E*). However, in contrast to previous observations with *FXN* KO cells, we found that lipoylation was not rescued under low oxygen tensions in *FDX1* KO cells (Fig. 1, *D* and *E*) (23). These results indicate that the lipoylation is neither restored by hypoxia nor required for the growth of *FDX1* KO cells in hypoxia.

### Proteomic profiles of FXN, FDXR, FDX1/2, and LIAS KO cells in normoxia and hypoxia

We next sought to use a more global approach to get a sense of how FDX1 and FDX2 differentially affect protein expression in normoxia and hypoxia. We performed quantitative proteomics in HepG2 cells grown in normoxia and hypoxia to (i) gain a broad and systematic understanding of the downstream cellular changes induced by loss of either FDX, (ii) to explore the relationship between FDX1 and the lipoate synthesis pathway, and (iii) to define the proteomic responses to hypoxia. Alongside control, *FDX1*, *FDX2*, and *FDXR* KO cells, we also chose to study *FXN* KO cells to compare our FDX datasets with cells suffering a deficiency of ISC synthesis that can be rescued by hypoxia, as well as *LIAS* KO cells to compare with cells affected by a defect in the lipoate synthesis pathway. The KO cells were confirmed by immunoblot analysis (Fig. S4) (28, 29).

**Figure 2:**
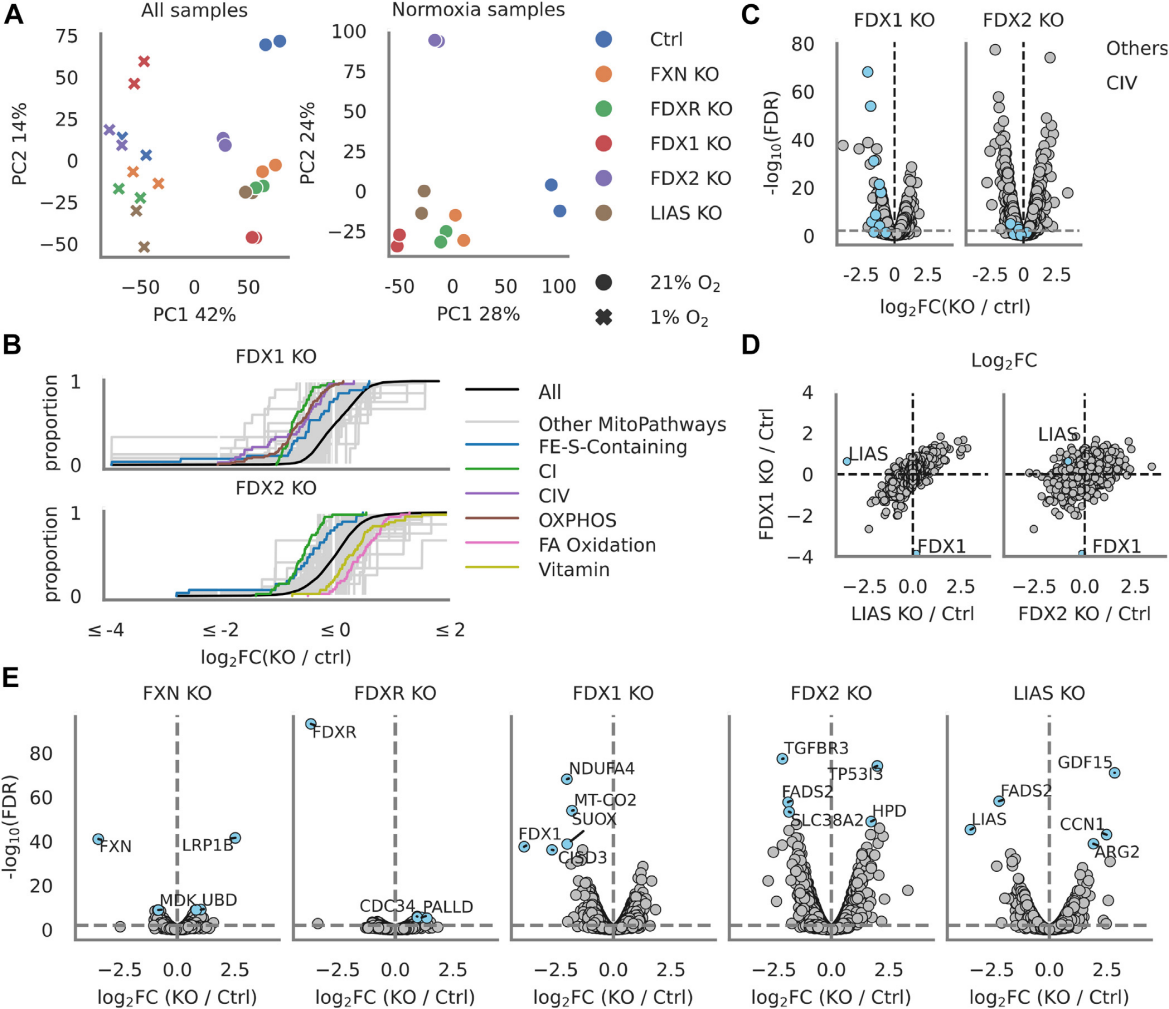
**Proteomics of HepG2 KO cells highlights divergent roles for FDX1 and FDX2.***A*, principal component analysis (PCA) of 7692 proteins detected in duplicate HepG2 cell samples edited with control (CTRL), *FXN*, *FDXR*, *FDX1*, *FDX2*, or *LIAS* guides grown in 21% (normoxia) or 1% O_2_ (hypoxia). Principal components calculated for all samples together or normoxic samples separately. *B*, cumulative distribution functions of 149 MitoCarta MitoPathways in *FDX1* or *FDX2 KO* samples compared with controls in normoxia. Labeled are those pathways achieving a false discovery rate (FDR) <0.0002. *C*, volcano plots highlighting log_2_ fold changes and corresponding FDR for all proteins in *FDX1* and *FDX2* KO samples compared with controls in normoxia. Complex IV subunits are shown in *blue*; *gray horizontal line* denotes FDR = 0.01 *D*, distribution of log_2_ fold changes for all proteins in *FDX1*, *FDX2*, and *LIAS* KO samples compared with controls in normoxia. *E*, volcano plots depicting log_2_ fold changes and corresponding FDR for all proteins in all KO samples compared with controls in normoxia. Selected proteins with significant FDR are highlighted in *blue*. *Gray horizontal line* denotes FDR = 0.01. FDX, ferredoxin.

In total, we could quantify the abundance level of 7692 proteins in *FDX1*, *FDX2*, *FDXR*, *LIAS*, *FXN*, and control KO HepG2 samples in duplicate across the two oxygen tensions (Table S1). Principal component analysis revealed strong separation of samples by oxygen tension (principal component 1, explaining 42% of variance) (Fig. 2*A*). When we focused this analysis only on the normoxic samples, we found that *LIAS* and *FDX1* KO samples clustered closely together (along with *FXN* and *FDXR* KOs), whereas *FDX2* KO samples segregated from the rest (Fig. 2*A*).

### FDX1 and FDX2 KO exhibit distinct proteomic profiles

Our analyses suggest very different proteomic responses to FDX1 *versus* FDX2 loss. We considered the impact of these knockouts on all mitochondrial pathways, using an inventory of 149 MitoPathways from MitoCarta3.0 (39). We plotted the cumulative distribution of log fold changes caused by each KO when compared with the control KO in normoxia for all proteins as well as for those in each mitochondrial pathway, where pathways with a false discovery rate (FDR) <0.0002 are colored (Fig. 2*B*). Of note, larger proportions of proteins involved in mitochondrial pathways were downregulated compared with the overall change in protein expression level in *FDX1* but not *FDX2* KO cells (Fig. 2*B*).

CI subunits and ISC-containing proteins were amongst the most significantly downregulated pathways in both knockouts, whereas fatty acid oxidation and vitamin metabolism were enriched specifically with FDX2 loss (Fig. 2*B*). Consistent with our immunoblot analysis, we also saw CIV as a significantly depleted pathway in *FDX1* but not *FDX2* KO samples (Fig. 2*B*).

To further understand whether the downregulation of the CIV pathway in *FDX1* KO cells was attributable to a few proteins with dramatic negative changes in their expression levels, or to a general depletion of all CIV-associated peptides, we examined both log fold change and significance of differential protein expression in *FDX1* and *FDX2* KO samples compared with controls in normoxia and focused on the distribution of proteins annotated in the CIV pathway (Fig. 2*C*). We saw a clear significant depletion of almost all detected CIV-associated proteins (shown in *blue*) in *FDX1* but not *FDX2* KO samples (Fig. 2*C*). This result agrees with our immunoblot studies and recent reports that implicate FDX1 in heme A synthesis (36). Our proteomics observations further solidify the idea that the heme A synthesis pathway has been misannotated and is likely attributable to FDX1 not FDX2.

Although FDX1 and FDX2 are sequence paralogs (17), the proteomic and immunoblot analyses suggest divergent function. We therefore tested if gentle overexpression of FDX2 could functionally complement lipoate deficiency in *FDX1 KO* cells. We analyzed growth and lipoate production of control and *FDX1* KO cells that were simultaneously overexpressing GFP, FDX2, or a guide-resistant FDX1 complementary DNA (cDNA) (Fig. S5, *A* and *B*). We confirmed localization of these constructs by analyzing protein collected from whole cell and mitochondrial lysates (Fig. S5*C*). We found that only when the KO cells overexpressed guide-resistant FDX1 did they recover growth or lipoate production (Fig. S5, *A* and *B*). We conclude that the two FDXs cannot substitute for one another in lipoate synthesis, consistent with a recent study reporting that only FDX1 donates electrons for LIAS SAM catalysis (36).

### FDX1 KO and LIAS KO display similar proteomic profiles

Because we saw closer clustering of *FDX1* and *LIAS* KO samples to each other in normoxia compared with other knockouts, we visualized the log fold changes of proteins in *FDX1*, *FDX2*, and *LIAS* KO samples compared with controls in normoxia (Fig. 2*D*). The scatter plots revealed a linear correlation between *FDX1* and *LIAS* KO samples and not between the *FDX1* and *FDX2* KO samples (Fig. 2*D*). These results indicate that LIAS and FDX1 loss result in similar changes across the proteome, whereas the consequences of FDX1 loss and FDX2 loss are clearly more distinct.

In addition, we analyzed the differential protein expression across all knockouts in normoxia and labeled those proteins with the most significant changes in each knockout (Fig. 2*E*). We noted that the spread of the significance level of protein differential expressions provided a broad view of how loss of each protein was affecting the HepG2 cellular proteome, with FXN and FDXR ablation clearly having few significant effects, FDX1 and LIAS loss producing similar ranges of significance to each other, and FDX2 loss causing the most dramatic “eruption” in number of proteins differentially expressed with high significance (Fig. 2*E*).

The lack of significant proteomic responses to FDXR loss was surprising. It is unknown whether this arises from incomplete ablation of the protein because FDXR has many isoforms (40), though our proteomics analysis reveals that the target protein is strongly depleted. Alternatively, an unknown oxidoreductase could compensate for FDXR loss. Other striking and notable changes include the strong increase in GDF15 (a marker of the integrated stress response) (41, 42) with *LIAS* deletion and the significant depletion in sulfite oxidase abundance with loss of FDX1 (Fig. 2*E*) (43).

### LIAS requires both ISC-binding sites for stability

**Figure 3:**
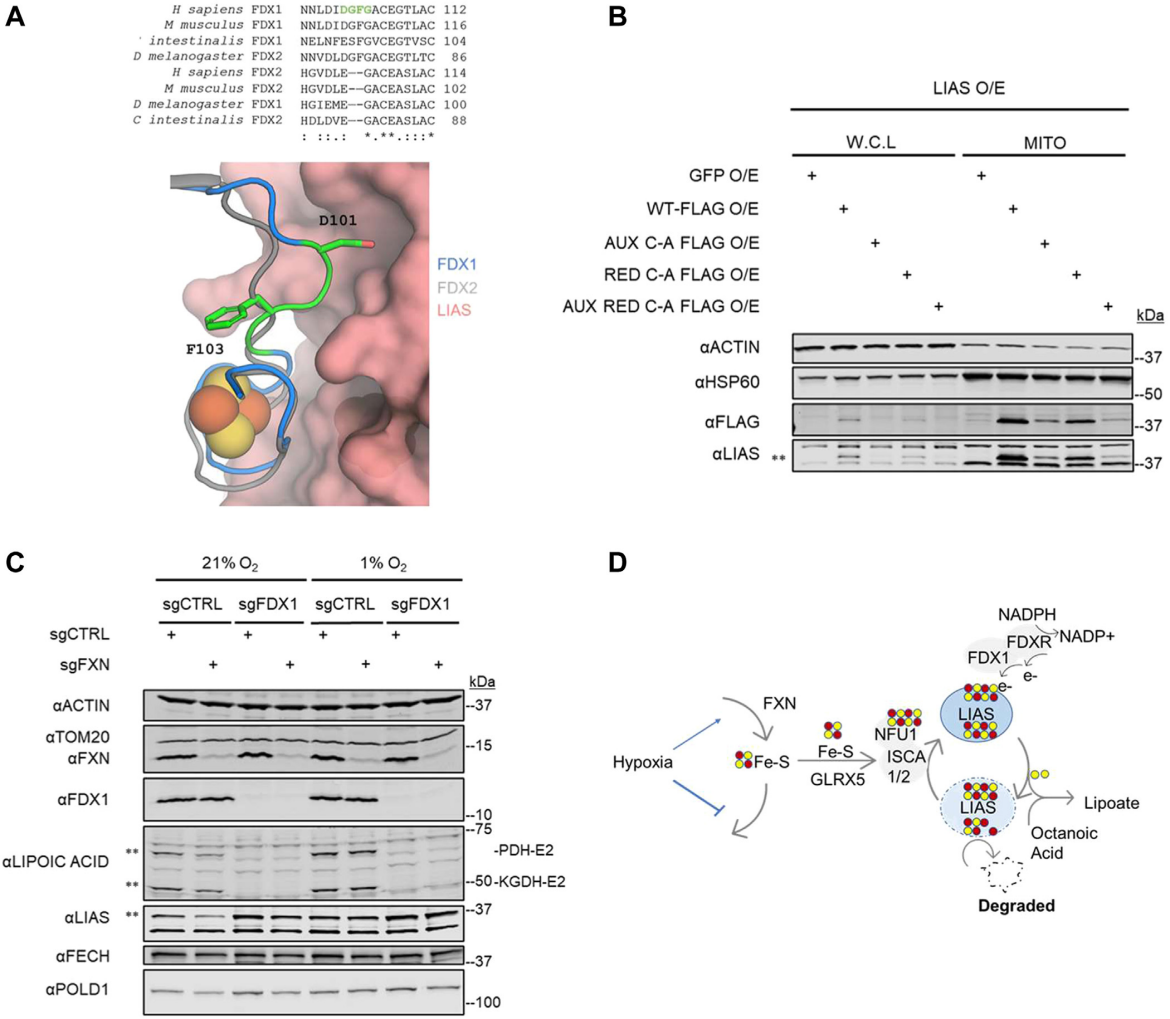
**FDX1 loss stabilizes LIAS in an ISC-depleted cell.***A*, *top*, a multiple sequence alignment of FDX1 and FDX2 homologs from several eukaryotic organisms, highlighting key FDX1 residues in *green* that are absent in FDX2 (N.B., nomenclature for FDX1 and FDX2 is inverted in *Drosophila melanogaster* compared with other eukaryotes). *Bottom*, the top ranked interaction model from AlphaFold analysis of LIAS (*pink*, surface) and FDX1 (*blue* and *green*, *cartoon*). FDX2 (g*ray*, *cartoon*) has been structurally aligned with FDX1. The *green residues* in FDX1 that are divergent in FDX2 form part of the interface with LIAS. *B*, immunoblots for FLAG, LIAS, and control proteins HSP60 and ACTIN on whole cell lysate (W.C.L) and isolated mitochondrial (MITO) lysates of K562 cells overexpressing (O/E) GFP or four different LIAS constructs with 1× FLAG tags on the C-terminal end. Constructs expressed were either WT LIAS or LIAS with cysteine to alanine mutations in either the auxiliary cluster site (AUX C-A FLAG), the reducing cluster site (RED C-A FLAG), or both the auxiliary and reducing cluster site (AUX RED C-A FLAG). *C*, immunoblots for FXN, FDX1, LIAS, FECH, POLD1, lipoylated PDH and KGDH, and control proteins ACTIN and TOM20 on lysates from K562 cells edited with control (CTRL) or *FXN* guides on the background of prior editing with control (CTRL) or *FDX1* guides. *D*, proposed model of LIAS turnover in the absence of FDX1. If turnover of the LIAS enzyme is halted by eliminating FDX1, then LIAS is no longer dependent on the ISC pool for its stability. *Double asterisk* indicates band of interest. FDX, ferredoxin; ISC, iron–sulfur cluster; LIAS, lipoyl synthase.

Our proteomics observations that FDX1 loss phenocopies LIAS loss led us to initially suspect that the mechanism of lipoate depletion in *FDX1* KO cells could be due to destabilization of LIAS. However, our proteomics indicated that LIAS abundance actually rises in *FDX1* KO cells, consistent with the protein being stabilized by FDX1 loss (Fig. 2*D*). LIAS is a radical SAM enzyme with two 4Fe–4S clusters (reducing and auxiliary) (29, 44, 45). Protein modeling and sequence alignment studies from our laboratory and others indicate FDX1 (but not FDX2) interacts with LIAS at the site of the reducing ISC (Figs. 3*A* and S6) (36). FDX1 donates electrons to this reducing cluster (36), creating a radical species that activates the octanoate precursor that then abstracts sulfur atoms from the auxiliary cluster to form the mature lipoate (29, 36, 45, 46). Prior studies have shown that the regeneration of the auxiliary cluster is important for the stability of LIAS (29, 44, 45), and therefore, cells with defective mitochondrial ISC synthesis or trafficking have low LIAS levels (34, 35, 38). We evaluated the impact of mutating one or both ISC-binding sites on LIAS protein stability by overexpressing different mutant constructs of the protein in WT K562 cells. We engineered and provided cells with cDNA of either a (i) GFP control, (ii) WT LIAS, (iii) auxiliary ISC-binding site mutant (aux C→A), (iv) reducing ISC-binding site mutant (red C→A), (v) or a double mutant of both reducing and auxiliary ISC-binding sites (aux C→A, red C→A) (Fig. 3*B*). Expression of these constructs was well tolerated (Fig. S7), and immunoblot analysis on whole cell and mitochondrial lysates indicated that both mutants resulted in decreased LIAS stability, though the auxiliary site was more critical. Mutation of both sites resulted in an almost complete loss of the protein (Fig. 3*B*). Because loss of FDX1 did not result in loss of LIAS protein (Fig. 2*D*), it is unlikely that ISCs on LIAS are destabilized by FDX1 loss.

### FDX1 and FXN loss have opposing effects on LIAS stability

During each catalytic cycle, the auxiliary cluster on LIAS loses two sulfurs that must be reloaded, and therefore, LIAS is dependent on an adequate supply of ISCs (29, 34, 38, 45). We were curious if this dynamic was affected by FDX1 loss. We edited K562 cells with control or FDX1 guides and then simultaneously used control or *FXN* guides to induce ISC depletion. Cells were then grown in normoxia and hypoxia and collected for immunoblotting. As expected, we saw that LIAS levels were depleted with FXN loss (likely because of loss of the auxiliary ISC) and stabilized with FDX1 loss (Figs. 3*C* and S8). However, LIAS levels trend toward being restored in *FXN/FDX1* double KOs (Figs. 3*C* and S8) (*p* = 0.16), suggesting that the additional KO of *FDX1* restored LIAS protein in a *FXN* KO background. We validated that the restorative phenotype was not caused by a general buffering of ISC loss by confirming that other ISC proteins FECH and POLD1 were still depleted in *FDX1/FXN* double KO cells (Fig. 3*C*). We in addition observed that the lipoate depletion in *FXN/FDX1* double KO cells was not rescued under low oxygen (Fig. 3*C*), indicating hypoxia could no longer restore lipoate in an *FXN* KO cell when FDX1 was absent. In addition, LIAS levels under all conditions were similarly boosted by hypoxia (Figs. 3*C* and S8). Our results are consistent with a model whereby FDX1 loss prevents the LIAS enzyme from achieving a catalytically active state that leads to ISC loss (Fig. 3*D*).

### Loss of lipoate synthesis is tolerated in multiple cell types under hypoxia

**Figure 4:**
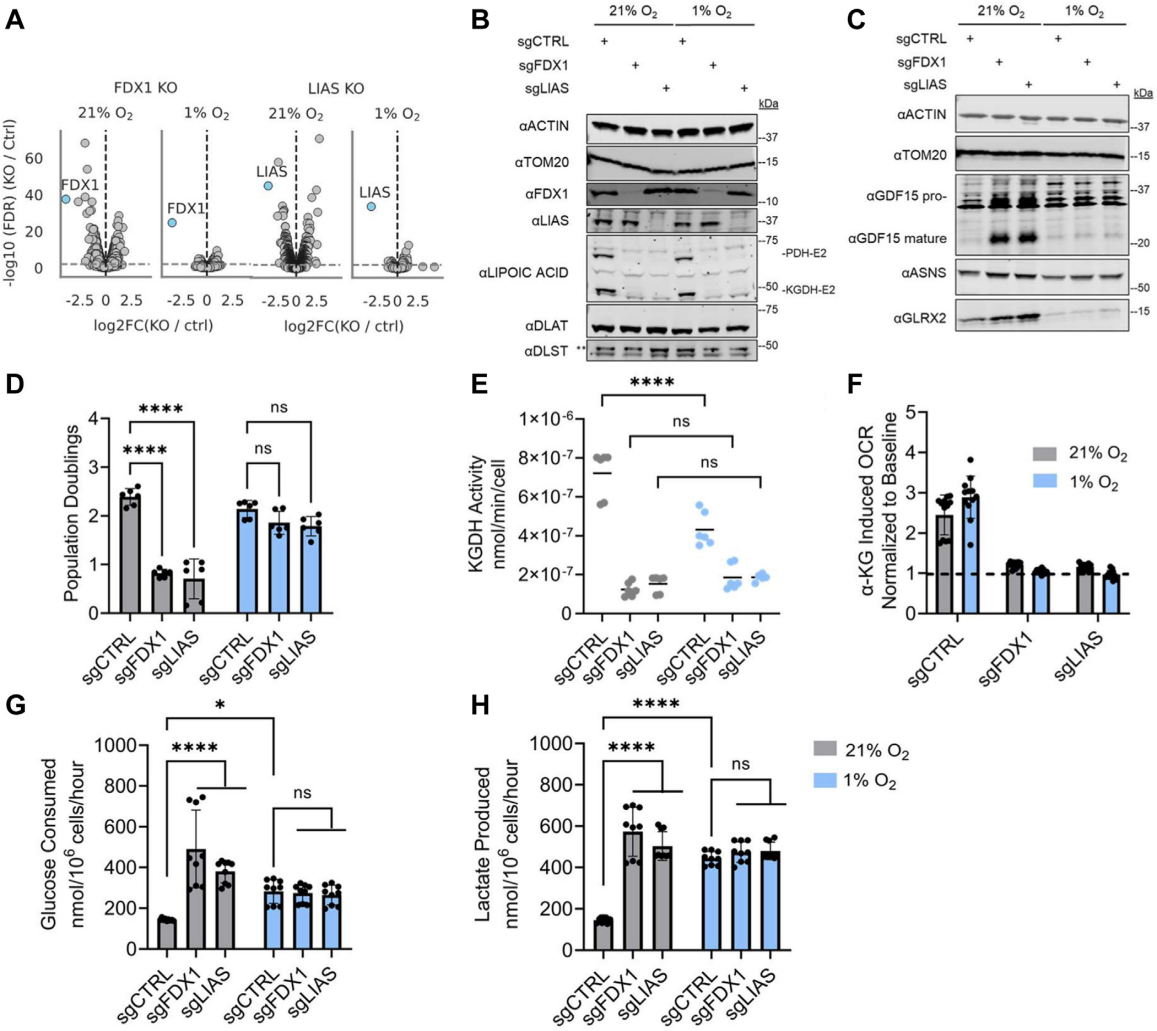
**Lipoate synthesis is dispensable under low oxygen tensions.***A*, volcano plots depicting log_2_ fold changes and corresponding FDR for all proteins in *FDX1* and *LIAS* KO samples compared with controls in normoxia (21% O_2_) and hypoxia (1% O_2_). FDX1 and LIAS are shown in *blue*, *gray horizontal line* denotes FDR = 0.01. *B*, immunoblots for FDX1, LIAS, lipoylated PDH and KGDH, E2 subunit proteins of PDH (DLAT) and KGDH (DLST) enzyme complexes, and control proteins ACTIN and TOM20 on lysates of HepG2 cells edited with control (CTRL), *FDX1*, or *LIAS* guides and grown in 21% or 1% O_2_. *Double asterisk* indicates band of interest. *C*, immunoblots for GDF15, ASNS, GLRX2, and control proteins ACTIN and TOM20 on lysates of HepG2 cells edited with control (CTRL), *FDX1*, or *LIAS* guides and grown in 21% or 1% O_2_. *D*, three-day proliferation assay of HepG2 cells edited with control (CTRL), *FDX1*, or *LIAS* guides and grown in 21% or 1% O_2_. *E*, KGDH activity assayed by a KGDH enzyme activity kit using HepG2 cells edited with control, *FDX1*, or *LIAS* guides and grown in 21% or 1% O_2_. *F*, bar plot displaying fold change in oxygen consumption rate (OCR) as assessed *via* permeabilized cell seahorse assays run at 21% and 1% O_2_ on HepG2 cells edited with control (CTRL), *FDX1*, or *LIAS* guides. Oxygen consumption rates following injection of α-KG were normalized to baseline readings per well. *Dashed line* indicates normalized baseline. *G*, YSI measured glucose consumption over a 3-day period in HepG2 cells edited with control (CTRL), *FDX1*, or *LIAS* guides and grown in 21% or 1% O_2_, normalized to final cell count on day 3. *H*, YSI measured lactate production over a 3-day period in HepG2 cells edited with control (CTRL), *FDX1*, or *LIAS* guides and grown in 21% or 1% O_2_, normalized to final cell count on day 3. All bar plots show mean ± SD of three independent experiments. ns = *p* > 0.05, ∗*p* ≤ 0.05, ∗∗*p* ≤ 0.01, ∗∗∗*p* ≤ 0.001, ∗∗∗∗*p* ≤ 0.0001. Two-way ANOVA with Bonferroni’s post-test. α-KG, α-ketoglutarate; FDR, false discovery rate; KGDH, α-ketoglutarate dehydrogenase; LIAS, lipoyl synthase; PDH, pyruvate dehydrogenase.

Given the shared proteomic signatures between *FDX1* and *LIAS* KO cells in normoxia (Fig. 2, *A* and *D*), we next analyzed the proteomes of these KO cells in hypoxia. Volcano plots reveal striking changes in the proteome following FDX1 loss in normoxia, but these “eruptions” were attenuated in hypoxia (Fig. 4*A*). We observe a similar pattern in *LIAS* KO cells, where many proteins are differentially expressed with high levels of significance in 21% oxygen but not in 1% oxygen (Fig. 4*A*).

We validated some of the key proteomic changes by immunoblot analysis. Again, we confirmed our earlier observation with *FDX1* KO cells (Fig. 1, *D* and *E*) that lipoylation was not rescued by hypoxia in *LIAS* KO cells (Fig. 4*B*). Immunoblot analysis validated our proteomics results, and markers of the integrated stress response (GDF15, ASNS) (41, 42, 47), and oxidative stress (GLRX2) (48), were rescued by hypoxia (Fig. 4*C*). In addition, both genetic KOs were tolerated under hypoxic conditions, in that their growth improved with exposure to low oxygen and was no longer significantly different from the control KO cells (Fig. 4*D*). We further confirmed this growth phenotype in K562 cells (Fig. S9*A*) and validated in both cell lines that depletion of signal in our lipoate immunoblots was not attributable to any downstream loss of the E2 subunits in PDH (DLAT) or KGDH (DLST), which are modified with the lipoate cofactor (Figs. 4*B* and S9*B*) (27).

We used two different methods to interrogate the activity of lipoate-containing KGDH, in our HepG2 *FDX1* and *LIAS* KO cell lines in normoxia and hypoxia. (Fig. 4*E*). First, we performed a KGDH activity assay using extracts from control and KO cells grown in normoxia and hypoxia. The activity of KGDH was lost in *FDX1* and *LIAS* KO cells and was not rescued by hypoxia (Fig. 4*E*). Second, we performed permeabilized cell seahorse experiments and found that feeding α-ketoglutarate (α-KG) to *FDX1* KO or *LIAS* KO cells did not result in an increase in oxygen consumption rate over baseline regardless of ambient oxygen tension (Fig. 4*F*).

In the absence of lipoate-containing enzymes, a shift toward glycolytic metabolism is expected (28, 49, 50). Indeed, *FDX1* KO and *LIAS* KO cells consumed more glucose and produced more lactate in normoxia relative to our control cells (Fig. 4, *G* and *H*). Under hypoxia, *FDX1* and *LIAS* KO cells exhibited rates of glucose consumption and lactate production equivalent to those of control cells (Fig. 4, *G* and *H*). However, the KO cells did not consume more glucose or produce more lactate in hypoxia than in normoxia, whereas control KO cells did increase their glucose consumption and lactate production under low oxygen tensions, as expected (Fig. 4, *G* and *H*) (31, 33, 49).

Collectively, these results confirm that the lipoate-containing enzyme KGDH is not able to function without lipoylation of its E2 subunit, and that in the absence of lipoate, cells shift from oxidative phosphorylation to glycolysis both in normoxia and hypoxia. Although the mechanism by which hypoxia is allowing cells to tolerate loss of lipoylation is currently not known, it does not appear to be due to a simple shift from oxidative metabolism to glycolysis as the KO cells achieve this even in normoxia.

## Discussion

Here, we have explored the functions of the mitochondrial FDXs and their requirements in hypoxia, and in the process, have discovered a key role for FDX1 as a partner for LIAS in lipoic acid production. Surprisingly, we find that loss of FDX1 and LIAS, as well as lipoylation, is all tolerated in two different human cell types grown in hypoxic conditions.

Our discovery that FDX1 is required for the lipoate synthesis pathway is richly supported by two recent reports (26, 36) as well as other contemporary work (51). Here, we confirm reports that FDX1 is required for lipoate synthesis, and that knocking it out stabilizes the enzyme LIAS (26, 36). Using a genetic strategy, we find that FDX1 is upstream of LIAS and that the loss of FDX1 stabilizes LIAS protein in cells with reduced ISC synthesis, possibly by preventing the LIAS enzyme from entering its catalytic cycle (Fig. 3, *C* and *D*). The catalytic cycle of LIAS involves an offloading and reloading of the auxiliary 4Fe–4S cluster (29, 36). In the absence of FXN, the deficiency of ISCs leads to a destabilization of LIAS (Fig. 3, *C* and *D*). FDX1 activity is necessary to initiate LIAS transition to an unstable state until the ISC is replenished. When FDX1 is missing, LIAS is no longer undergoing turnover and is stabilized (Figs. 3, *C* and *D* and S8).

Our proteomics analysis identifies cellular pathways that appear to lie downstream of the mitochondrial FDXs. Of note, sulfite oxidase (a Moco-dependent enzyme in sulfur metabolism) (42) was uniquely depleted upon loss of FDX1 (Fig. 2*E*) and would be potentially interesting for further exploration. We are also intrigued by the modest effect of FDXR loss in HepG2 cells in normoxia (Fig. 2*E*). The differences between *FDXR* KO and *FDX1* or *FDX2* KO cells strongly call into question the idea that FDXR is the sole electron donor for this pathway, as loss of either downstream FDX causes larger proteomic changes than loss of the reductase (Fig. 2*E*) (8). These data could imply the availability of alternative electron donors that can complement the need for FDXR function or that very low levels of FDXR may persist because of incomplete genetic ablation of alternative transcripts that are not detected by our proteomics (40). We also complete the validation of all members of the ISC machinery and confirm that only FXN is fully dispensable under low oxygen tensions (with FDXR rescue being somewhat cell line specific) (Figs. 1, *B* and *C* and S2*A*) (23). The reasons underlying this unique shared property for these specific proteins remain unknown.

One of the most surprising findings from our work is that in multiple cell lines, loss of lipoate can be tolerated in hypoxia (Figs. 4, *B* and *D* and S9, *A*, and *B*). Lipoate is an ancient cofactor found in all three domains of life and in humans is critical for the function of key TCA cycle enzymes including PDH (28, 52). Previous work in *Escherichia coli* has shown that expression of the oxygen-labile enzyme pyruvate formate lyase is sufficient to maintain viability without lipoate in anaerobic conditions (52, 53). Some bacteria and parasitic organisms are also able to salvage lipoate from the media (27, 54, 55, 56). In *Saccharomyces cerevisiae*, previous reports have shown that mutants with defects in lipoate synthesis can grow on fermentative media but not on respiratory media (38, 57). In the current work, we find that lipoylation is almost completely ablated with loss of either FDX1 or LIAS in both oxygen tensions, arguing against the existence of alternative biosynthetic routes or salvage pathways (Fig. 4*B*). In our prior low/high O_2_ CRISPR screen, many additional genes encoding proteins required for lipoate synthesis also scored, including members of type II fatty acid synthesis pathway (required to generate the octanoate precursor for lipoate) (24, 27, 37), NFU1 (necessary for repair of the auxiliary cluster on LIAS), and BOLA3 (the putative assembly factor of LIAS) (24, 35, 38, 58). It is notable that multiple components of PDH, including DLAT, the lipoate-containing subunit, also scored highly in that screen (24, 27, 59).

Our results indicate that for cells lacking FDX1 or LIAS, growth in hypoxia continues despite the absence of lipoylation. Although the mechanism is unknown, it does not appear to be a simple buffering by hypoxia-driven increase in glycolysis because even in normoxia, the KO cell lines achieve a definitive shift toward glycolysis (Fig. 4, *G* and *H*). Hypoxia appears to create a state that is tolerant of lipoate deficiency, as stress responses evident in the proteome are restored to control levels (Fig. 4*C*). Future studies will be required to determine how hypoxia allows cells to tolerate loss of lipoylation. Mutations in various components of the lipoate synthesis pathway (including LIAS) are associated with debilitating mitochondrial diseases (60). It will be interesting to determine whether these conditions are exacerbated by hyperoxia—as we have shown for CI deficiency or FXN deficiency—and conversely, whether they may benefit from “hypoxia therapy (23, 61).”

## Experimental procedures

### Data analysis

All bar plots were analyzed by two-way ANOVA with Bonferroni’s post-test, with a threshold of *p* ≤ 0.05. All data were analyzed in PRISM (GraphPad Software, Inc) with the appropriate multiple comparisons, and all immunoblots were analyzed initially and exported from ImageStudio (LI-COR Biosciences). Each graphed dot in bar plots represents a single data point.

### Cell lines and culturing

K562 (female), human embryonic kidney 293T (female), and HEPG2 (male) cells were obtained from American Type Culture Collection and cultured in Dulbecco's modified Eagle's medium (DMEM) (Gibco) with 25 mM glucose, 10% fetal bovine serum (nondialyzed; Invitrogen), 4 mM glutamine, 1 mM sodium pyruvate, 50 μg/ml uridine, and 100 U/ml penicillin/streptomycin under 5% CO_2_ at 37 °C. Cell lines were checked by American Type Culture Collection profiling before purchase. Cells were tested to ensure the absence of mycoplasma by PCR-based assay once every 3 months. Cells were passaged every 2 to 3 days. Adherent cells were washed with PBS (Invitrogen) and dissociated using TrypLE (Gibco). For experiments involving hypoxia, cells were placed in Coy O_2_ control dual hypoxia chambers maintained at 37 °C, 1% O_2_, and 5% CO_2_ with appropriate humidity control. Cells were treated with 75 μM FG-4592 (1:1000 dilution from a 75 mM stock) (Selleck Chemicals) or dimethyl sulfoxide (Fisher Scientific).

Individual single-guide RNAs were cloned into pLentiCRISPRv2 (Addgene; catalog no.: 52961) (62), containing a puromycin or a hygromycin selection cassette. For studying growth of FXN/FDX1 double KO cells, K562 cells previously infected with prDA_186 (Addgene; catalog no.: 133458), bearing guides against a control locus or FXN, were used for some replicates, whereas repeated infection using guides with two different selection cassettes were used for others. For overexpression assays, cDNAs were either purchased from ORIGENE or custom synthesized from IDT. When necessary, 1× FLAG or 1× FLAG + 1× MYC tags were added to the C terminus. Constructs were cloned in pLYS6 bearing a neomycin selection cassette, using the NheI and EcoRI sites. All plasmids were verified by sequencing. pMD2.G (Addgene; catalog no.: 12259) and psPAX2 (Addgene; catalog no.: 12260) were used for lentiviral packaging.

### Lentivirus production

About 2.5 × 10^6^ or 6.25 × 10^6^ human embryonic kidney 293T cells were seeded in 5 or 10 ml in a T25 cm^2^ flask or a 10 cm dish (one lentivirus per flask). The following day, the cells were transfected with 1 (or 2) ml of transfection mixture. The transfection mixture contained 25 (or 50) μl Lipofectamine 2000 (Thermo Fisher Scientific), 3.75 (7.5) μg psPAX2, 2.5 (5) μg pMD2.G, 5 (10) μg of lentiviral vector of interest, and Opti-MEM medium (Gibco) up to 1 (2) ml. The mixture was incubated at room temperature (RT) for 20 min before adding it to cells. Six hours following transfection, the media were replaced with fresh DMEM. Two days after transfection, media were collected, filtered through a 0.45 μM filter, and stored at −80 °C.

### Infection

K562 cells were seeded at 5 × 10^5^ cells/ml in 2 ml per well in a 6-well plate the day of infection. Cells were infected with virus, and polybrene was added at 1:1000 final volume (Invitrogen). Cells were incubated overnight before being selected with puromycin (2 μg/ml final concentration) (Gibco), geneticin (500 μg/ml) (Gibco), or hygromycin (Gibco) (250 μg/ml) for 48 h.

HepG2 cells were seeded at 6 × 10^5^ cells per well the day before infection in a 6-well plate. Cells were then infected with virus, and polybrene was added at 1:2000 final volume (Invitrogen). Cells were incubated overnight before being selected with puromycin (3 μg/ml final concentration) (Gibco) for 48 h.

### Polyacrylamide gel electrophoresis and protein immunoblotting

About 2 to 5 × 10^6^ K562 or 3 to 6 × 10^6^ HepG2 cells were harvested, washed in cold PBS, and lysed for 10 to 25 min on ice in radioimmunoprecipitation lysis buffer (Thermo Fisher), 1× HALT protease and phosphatase inhibitor (Thermo Fisher), and Pierce Universal Nuclease for Cell Lysis (Thermo Fisher). Lysates were further clarified by centrifugation for 10 min at 10,000*g* at 4 °C. Supernatant was collected into fresh tubes, and protein concentration was measured with the Pierce 660 nm protein assay (Thermo Fisher). About 30 μg was loaded per well in Novex Tris–glycine 4 to 20% or 10 to 20% gels (Life Technologies). Gels were run for 50 min at 200 V and transferred onto a nitrocellulose membrane, 0.45 μM (Bio-Rad). Membranes were stained with Ponceau S to check for adequate loading. Membranes were then blocked for 1 to 2 h with Odyssey Blocking Buffer (LI-COR Biosciences) at RT. Afterward, membranes were incubated overnight at 4 °C with a solution of primary antibody diluted in 3% bovine serum albumin in Tris-buffered saline with Tween-20 (TBS-T) + 0.05% N_3_. The next day, membranes were washed at RT five times in TBS-T for 5 min. The membrane was incubated with goat anti-rabbit or antimouse conjugated to IRDye800 or IRDye680 (LI-COR Biosciences) in a 1:1 solution of Odyssey blocking buffer (LI-COR Biosciences) and TBS-T. Membranes were incubated for 1 h at RT and then washed three times in TBS-T for 10 min each. Membranes were then scanned for infrared signal using the Odyssey Imaging System (LI-COR Biosciences). Band intensities were analyzed with Image Studio LITE (LI-COR Biosciences).

**Table:** Antibodies

Antigen	Catalog number	Vendor
FXN	ab175402	Abcam
OXPHOS	Ab110411	Abcam
FDX1	12592-1-AP	ProteinTech
FDX2	HPA043986	Atlas
LIAS	11577-1-AP	ProteinTech
Tubulin	MA5-16308	Invitrogen
Actin	Ab8227	Abcam
Actin	8H10D10	Cell Signaling
FLAG	F3165-2MG	Sigma
DYKDDDDK Tag	2368	Cell Signaling
HSP60	ab45134	Abcam
TOM20	Sc-17764	Santa Cruz Biotechnology
FDXR	Sc-374436	Santa Cruz Biotechnology
Lipoic acid	437695	EMD Millipore
Lipoic acid	Ab58724	Abcam
POLD1	15646-1-AP	ProteinTech
FECH	14466-1-AP	ProteinTech
DLAT	12362	Cell Signaling
DLST	5556	Cell Signaling
GDF15	27455-1-AP	ProteinTech
ASNS	14681-1-AP	ProteinTech
GLRX2	13381-1-AP	ProteinTech

### Proliferation assays

Cell proliferation assays were performed between 8 and 10 days following lentiviral infection. Cells were seeded at an initial density of 1.5 × 10^5^ cells/ml (K562) or 2.5 × 10^5^ cells per well in a 6-well plate (HepG2) and cultured for 3 days in either 21% or 1% oxygen tensions. Viable cell numbers were then determined using a Vi-Cell Counter (Beckman).

### qPCR

About 2.5 to 3.5 × 10^6^ cells were collected per sample and snap frozen in liquid nitrogen and stored at −80 °C until use. Cells were then thawed on ice, and RNA was extracted using the QIAGEN RNeasy mini kit and DNASE-I digested before murine leukemia virus reverse transcription with random primers (Promega). qPCR was performed using TaqMan technology (Life Technologies) using probes HS00188949_m1 (BNIP3L) and HS00472881_m1 (PUM1).

### Proteomics

sgCTRL, sgFXN, sgFDXR, sgFDX1, sgFDX2, and sgLIAS HepG2 cells were grown for 6 days in 21% or 1% oxygen conditions in 150 mm plates. Cells were washed four times in ice-cold PBS, scraped into fresh ice-cold PBS, and spun down at 300*g* for 5 min at 4 °C in a microcentrifuge. The remaining PBS was siphoned off, and the cell pellets were snap frozen in liquid nitrogen and stored at −80 °C until the time of sample submission to the Thermo Fisher Scientific Center for Multiplexed Proteomics (Harvard).

#### Sample preparation for mass spectrometry

Samples for protein analysis were prepared essentially as previously described (63, 64). Following lysis, protein precipitation, reduction/alkylation, and digestion, peptides were quantified by micro–bicinchoninic acid assay and 100 μg of peptide per sample were labeled with TMTpro reagents (Thermo Fisher) for 2 h at RT. Labeling reactions were quenched with 0.5% hydroxylamine and acidified with TFA. Acidified peptides were combined and desalted by Sep-Pak (Waters).

#### Basic pH reversed-phase separation

Tandem mass tag (TMT)–labeled peptides were solubilized in 5% acetonitrile (ACN)/10 mM ammonium bicarbonate, pH 8.0, and 300 μg of TMT-labeled peptides was separated by an Agilent 300 Extend C18 column (3.5 μm particles, 4.6 mm ID and 250 mm in length). An Agilent 1260 binary pump coupled with a photodiode array detector (Thermo Scientific) was used to separate the peptides. A 45 min linear gradient from 10% to 40% ACN in 10 mM ammonium bicarbonate pH 8.0 (flow rate of 0.6 ml/min) separated the peptide mixtures into a total of 96 fractions (36 s). A total of 96 fractions were consolidated into 24 samples in a checkerboard fashion, acidified with 20 μl of 10% formic acid, and vacuum dried to completion. Each sample was desalted *via* Stage Tips and redissolved in 5% formic acid/5% ACN for LC-MS3 analysis.

#### Liquid chromatography separation and tandem mass spectrometry (LC–MS3)

Proteome data were collected on an Orbitrap Eclipse mass spectrometer (ThermoFisher Scientific) coupled to a Proxeon EASY-nLC 1200 LC pump (ThermoFisher Scientific). Fractionated peptides were separated using a 120 min gradient at 500 nl/min on a 35 cm column (i.d. 100 μm, Accucore, 2.6 μm, 150 Å) packed in-house. High-field asymmetric-waveform ion mobility spectrometry was enabled during data acquisition with compensation voltages set as −40, −60, and −80 V (65). MS1 data were collected in the Orbitrap (120,000 resolution; maximum injection time of 50 ms; automatic gain control [AGC] 4 × 10^5^). Charge states between 2 and 5 were required for MS2 analysis, and a 120 s dynamic exclusion window was used. Top 10 MS2 scans were performed in the ion trap with collision-induced dissociation fragmentation (isolation window of 0.5 Da; Turbo; normalized collision energy of 35%; maximum injection time of 35 ms; AGC 1 × 10^4^). An on-line real-time search algorithm (Orbiter) was used to trigger MS3 scans for quantification (66). MS3 scans were collected in the Orbitrap using a resolution of 50,000, normalized collision energy of 45%, maximum injection time of 200 ms, and AGC of 3.0 × 10^5^. The closeout was set at two peptides per protein per fraction (66).

#### Data analysis

Raw files were converted to mzXML, and monoisotopic peaks were reassigned using Monocle (67). Searches were performed using the Comet search algorithm against a human database downloaded from UniProt in May 2021. We used a 50 ppm precursor ion tolerance, 1.0005 fragment ion tolerance, and 0.4 fragment bin offset for MS2 scans. TMTpro on lysine residues and peptide N termini (+304.2071 Da) and carbamidomethylation of cysteine residues (+57.0215 Da) were set as static modifications, whereas oxidation of methionine residues (+15.9949 Da) was set as a variable modification.

Each run was filtered separately to 1% FDR on the peptide-spectrum match level. Then proteins were filtered to the target 1% FDR level across the combined dataset. For reporter ion quantification, a 0.003 Da window around the theoretical *m/z* of each reporter ion was scanned, and the most intense *m/z* was used. Reporter ion intensities were adjusted to correct for isotopic impurities of the different TMTpro reagents according to the manufacturer's specifications. Peptides were filtered to include only those with a summed signal-to-noise ≥120 across 12 TMT channels. The signal-to-noise measurements of peptides assigned to each protein were summed for a given protein. These values were normalized so that the sum of the signal for all proteins in each channel was equivalent, thereby accounting for equal protein loading.

Proteins that did not have a valid readout in any of the 24 channels were filtered out. To correct for differences caused by separate experiment runs, the python package pyComBat (version 0.3.2) was run on the log2-transformed data before projecting the corrected values back into linear space. Proteins with differential abundance across conditions were determined with the R package EdgeR (version 3.36.0) with exact testing, and the Benjamini–Hochberg multiple testing correction was applied to control for FDRs. Proteins with FDR of lower than 0.01 were considered to have significantly differential abundances between conditions. The downstream pathway enrichment analysis was completed using GSEA (version 4.2.3) PreRanked (68, 69) with a list of significant proteins ranked by their corresponding log2-fold changes as input. Candidate pathways for the enrichment analysis were taken from the Human MitoPathways 3.0 database (39).

### Protein modeling

Docking predictions between LIAS and FDX1 and FDX2 were obtained using ColabFold (https://github.com/sokrypton/ColabFold) through the AlphaFold2_mmseqs2 notebook (56, 57, 58). Runs were performed using pdb70 templates, alignments were through MMseqs2 in unpaired + paired mode, and num_recycles was set to 3. The top five models for each run were structurally compared between each other, and the predicted alignment error plots were used to assess the likelihood of the predicted interface (70, 71, 72).

### Mitochondria isolation

About 5 × 10^7^ cells were harvested, washed in PBS, and either snap frozen and stored in −80 °C before proceeding to the next step or washed immediately after with 10 ml MB buffer (210 mM mannitol, 70 mM sucrose, 10 mM Hepes–KOH at pH 7.4, 1 mM EDTA, and protease/phosphatase inhibitor). Cells were resuspended in 1 ml of MB buffer supplemented with 1× HALT protease phosphatase inhibitor (ThermoFisher Scientific) and transferred to 2 ml glass homogenizer (Kontes). Cells were broken with ∼35 strokes of a large pestle on ice. MB + protease/phosphatase was added up to 6 ml. The samples were then centrifuged at 2000*g* for 5 min, and the pellet was discarded. The supernatant was then centrifuged again at 13,000*g* for 10 min at 4 °C. The mitochondrial pellets were washed with MB buffer once and resuspended in radioimmunoprecipitation lysis buffer with protease inhibitor (1:100×) and universal nuclease (1:1000×).

### Enzyme activity assay

The KGDH enzyme activity kit was purchased from Sigma (MAK189). Sample processing and activity assays were carried out as per kit instructions. Briefly, 1 × 10^6^ cells were pelleted and washed once in PBS. Cells were then lysed in assay buffer for 10 min on ice in normoxia or hypoxia before being clarified by centrifugation at 4 °C for 5 min at 10,000*g*. The supernatant was collected, aliquoted, and snap frozen in liquid nitrogen before being stored in −80 °C for future use. On the day of the assay, samples were thawed on ice and aliquoted into a 96-well clear flat bottom plate (Corning). Kit-provided enzyme-specific developer and substrate reaction mixes were added to each sample, and the plate was then placed into a Cytation 5 instrument (BioTek). Absorbance was measured at 450 nm every minute at 37° for up to 2 h. Measurement analysis was then calculated as described in kit instructions.

### Permeabilized cell seahorse measurements

Oxygen consumption rate studies were conducted in a Seahorse XFe96 Analyzer at 21% or 1% O_2_ tensions. All experiments were conducted at 37 °C at pH 7.2. HepG2 cells were seeded the day before in standard media conditions in the provided seahorse cell culture microplate at 2.5 × 10^4^ cells/well. The Seahorse cartridge was hydrated with 200 μl per well of Seahorse XF Calibrant (Agilent) and placed in a 37 °C incubator overnight at either 21% or 1% oxygen. The following day, wash buffer, seahorse media, and injectable media were prepared in 1× MAS buffer (70 mM sucrose, 220 mM mannitol, 10 mM KH_2_PO_4_, 5 mM MgCl_2_, 2 mM Hepes, and 1 mM EGTA; pH 7.2). For hypoxia experiments, 1X MAS buffer was placed in the hypoxia glovebox overnight. About 10% fatty-acid free bovine serum albumin was added to both the wash buffer and seahorse media at a final concentration of 0.2%, and 0.5 M ADP was supplemented to the seahorse media at a final concentration of 4 mM along with 1 nM XF Plasma Membrane Permeabilizer (Agilent).Cells were washed twice with the wash buffer before being replated in Seahorse Media. After five to six baseline measurements, cells were injected with α-KG at a final concentration of 10 mM, followed by injections of oligomycin (4 μM final), and then piericidin/antimycin (5 μM final). For assays performed under hypoxia, edge wells on the plate were injected with 0.1 M sodium sulfite solution for oxygen tension calibration. For data analysis, the second measurement postinjection with α-KG (representative of three measurements) was divided by the last baseline measurement to normalize the data. Four technical replicates from each biological replicate were plotted in the bar plot.

### Glucose uptake and lactate release measurements

Glucose and lactate concentrations were measured using an automatic glucose and lactate analyzer YSI 2900 Series. Cells were seeded in 6-well dishes with 3 ml standard culture media as mentioned previously. At the end of 3 days, 500 μl of media were collected from each well and centrifuged at 300*g* in a microcentrifuge for 4 min to pellet any cell debris. About 200 μl of media were loaded onto a 96-well flat bottom clear plate (Corning). The YSI program read triplicate measurements from each well. Cells from each well were simultaneously counted using a Vi-Cell Counter (Beckman) to obtain final cell count. For data analysis, the glucose consumed values were subtracted from the established glucose values in the DMEM (25 mM). Lactate values were added to the known lactate values in the DMEM (0 mM). These concentrations were then divided by final cell count, the number of days for the experiment (3), and finally this corrected value was multiplied by the amount of media volume per well. Triplicate values from three biological replicates for each condition were plotted for the bar plots shown in the figure.

## Data availability

All data described within the article are contained in the document. The mass spectrometry proteomics data have been deposited to the ProteomeXchange Consortium *via* the PRIDE (73) partner repository with the dataset identifier PXD042589. Any further information and requests for resources and reagents should be directed to and will be fulfilled by the Lead Contact, Vamsi K. Mootha (vamsi@hms.harvard.edu).

## Supporting information

This article contains supporting information (70, 71, 72).

## Conflict of interest

V. K. M. is on the scientific advisory board of Janssen Pharmaceuticals and 5AM Ventures. V. K. M. is listed as an inventor on a patent application filed by Massachusetts General Hospital on the use of hypoxia as a therapy for mitochondrial and degenerative diseases.
