# Supplementary material for: Lipoylation is dependent on the ferredoxin FDX1 and dispensable under hypoxia in human cells
Source: J Biol Chem. 2023 Jul 20;299(9):105075. doi: 10.1016/j.jbc.2023.105075 (PMC10470009; doi:10.1016/j.jbc.2023.105075)
Supplement: Supporting Information [file mmc1.docx]

Supporting Information

Title: Lipoylation is dependent on the ferredoxin FDX1 and dispensable under hypoxia in human cells

Authors: Pallavi R. Joshi, Shayan Sadre, Xiayan A. Guo, Jason G. McCoy, Vamsi K. Mootha

S1 – qPCR relevant to Figure 1B

S2 – FDXR Growth Assay relevant to Figure 1C

S3 – Ponceau staining relevant to Figure 1E

S4 – Immunoblot of samples in Figure 2

S5 – Complementation assay described alongside Figure 2

S6 – Heat map data of model in Figure 3A

S7 – Growth assay of samples in Figure 3B

S8 – Densitometry analysis of blots in Figure 3C

S9- K562 Data confirming results in Figure 4B and 4D

Table S1 – Proteomics data described in Figure 2

S-1

**Figure S1**

Quantitative reverse-transcriptase PCR validation of the hypoxia-inducible transcript *BNIP3L* in K562 cells edited with control (CTRL), *FDX1*, *FDX2*, or *FDXR* guides and grown in 21% O_2_, 1% O_2_ or 21% O_2_+FG-4592. Gene expression values are relative to that of housekeeping gene *PUM1*. Bar plot shows mean ± SD of two independent experiments.

S-2

**Figure S2**

**A.** Three-day proliferation assay of HepG2 cells edited with control (CTRL) or *FDXR* guides and grown in 21% or 1% O_2_. **B**. Immunoblots for FDXR and control protein TOM20 on lysates of edited HepG2 cells used for proliferation assay. Bar plot shows mean ± SD of three independent experiments. ns = p > 0.05, * = p ≤ 0.05, ** = p ≤ 0.01, *** = p ≤ 0.001, **** = p ≤ 0.0001. Two-way ANOVA with Bonferroni’s post-test.

S-3

**Figure S3**

Ponceau S staining of a nitrocellulose membrane used for immunoblotting in Figure 1D. 30 ug of HepG2 samples edited with control (CTRL), *FDX1*, or *FDX2* guides and grown in 21% or 1% O_2_ were loaded onto a 4-20% polyacrylamide gel and resolved by gel electrophoresis.

S-4

**Figure S4**

Immunoblots for FXN, FDXR, FDX1, FDX2, LIAS, and control proteins ACTIN and HSP60 on lysates of edited HepG2 samples subjected to quantitative proteomics shown in Figure 2.

S-5

**Figure S5**

**A.** Three-day proliferation assay of K562 cells edited with control (CTRL) or *FDX1* guides and with low titer overexpression (O/E) of GFP, guide resistant FDX1-FLAG or FDX2-FLAG constructs. Cells were grown in 21% O_2_. **B.** Immunoblots for FLAG-tag, FDX1, FDX2, lipoylated PDH and KGDH, and control protein ACTIN on lysates of edited K562 cells used for proliferation assay. **C**. Immunoblots for FLAG-tag, FDX1, FDX2, and control proteins HSP60 and ACTIN on whole cell lysates (W.C.L) and enriched mitochondrial (MITO) lysates of K562 cells overexpressing GFP, FDX1-FLAG and FDX2-FLAG constructs. Double asterisks indicate bands of interest. Bar plot shows mean ± SD of three independent experiments. ns = p > 0.05, * = p ≤ 0.05, ** = p ≤ 0.01, *** = p ≤ 0.001, **** = p ≤ 0.0001. Two-way ANOVA with Bonferroni’s post-test.

S-6

**Figure S6**

AlphaFold generated heatmaps showing the domain position confidence for the top five ranked interaction models of FDX1 and LIAS, or FDX2 and LIAS. The x and y coordinates correspond to the expected position error at residue x if the true and predicted structures were aligned on residue y. Blue and red indicate a lower and higher position error respectively. The top right and bottom left sections of each plot correspond to the interdomain confidence between LIAS and the ferredoxin. The top ranked FDX1 LIAS complex has substantially lower interdomain position errors than the remaining FDX1 LIAS complex models or the FDX2 LIAS complex models (56,57,58).

S-7

**Figure S7**

Three-day proliferation assay in 21% O_2_ of K562 cells overexpressing (O/E) GFP, wild type LIAS (WT O/E), LIAS with four cysteine to alanine mutations in the auxiliary cluster site (AUX C-A O/E), LIAS with four cysteine to alanine mutations in the reducing cluster site (RED C-A O/E), or LIAS with four cysteine to alanine mutations in both the auxiliary and reducing cluster site (AUX RED C-A O/E), as seen in Figure 4A . All four LIAS constructs had a 1X FLAG tag on the C-terminal end. Bar plot shows mean ± SD of three independent experiments.

S-8


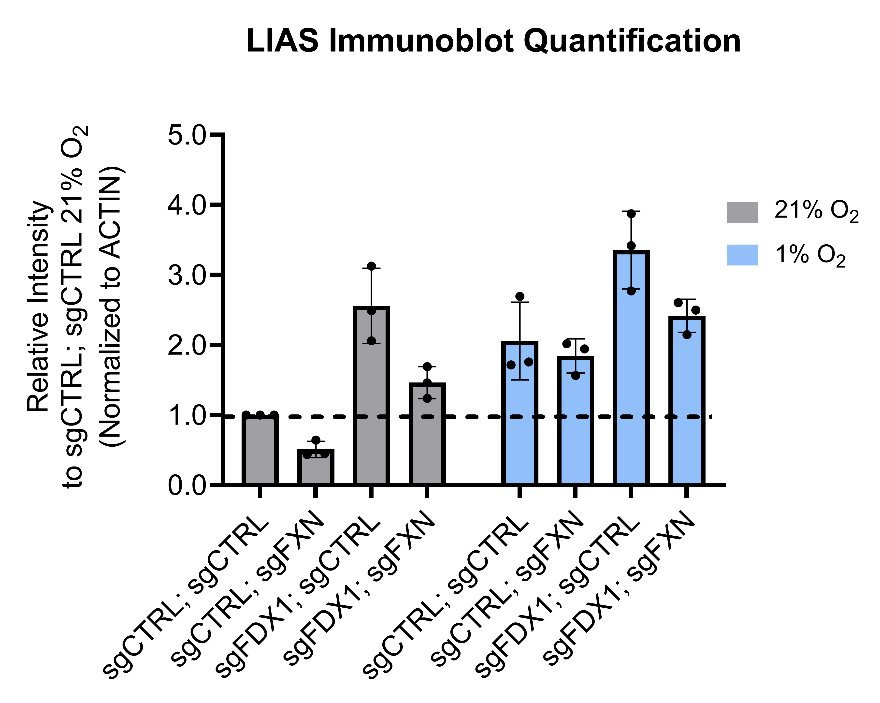


**Figure S8**

Densitometry analysis of LIAS band signal from immunoblots of K562 cells edited with control (CTRL) or *FXN* guides on the background of prior editing with control (CTRL) or *FDX1* guides and grown in 21% or 1% O_2_. Band intensity was quantified via ImageStudio and normalized to control protein ACTIN. Relative normalized intensity to bands from lysates of sgCTRL; sgCTRL cells in 21% O_2_ was calculated and plotted above. Dashed line indicates sgCTRL; sgCTRL 21% O_2_ normalized baseline. Bar plot shows mean ± SD of three independent experiments.

S-9

**Figure S9**

**A**. Three-day proliferation assay of K562 cells edited with control (CTRL), *FDX1*, or *LIAS* guides. Cells were grown in 21% O_2_ or 1% O_2_. **B**. Immunoblots for FDX1, LIAS, lipoylated PDH and KGDH, E2 subunit proteins of PDH (DLAT) and KGDH (DLST) enzyme complexes, and control proteins ACTIN and TOM20 on lysates of edited K562 cells used for proliferation assay. Double asterisk indicates band of interest. Bar plot shows mean ± SD of three independent experiments. ns = p > 0.05, * = p ≤ 0.05, ** = p ≤ 0.01, *** = p ≤ 0.001, **** = p ≤ 0.0001. Two-way ANOVA with Bonferroni’s post-test.
